# Anaerobic Growth of *Listeria monocytogenes* on Rhamnose Is Stimulated by Vitamin B_12_ and Bacterial Microcompartment-Dependent 1,2-Propanediol Utilization

**DOI:** 10.1128/mSphere.00434-21

**Published:** 2021-07-21

**Authors:** Zhe Zeng, Siming Li, Sjef Boeren, Eddy J. Smid, Richard A. Notebaart, Tjakko Abee

**Affiliations:** a Food Microbiology, Wageningen University and Research, Wageningen, The Netherlands; b Laboratory of Biochemistry, Wageningen University and Research, Wageningen, The Netherlands; University of Wisconsin—Madison

**Keywords:** *Listeria monocytogenes*, anaerobic catabolic pathways, microcompartment, rhamnose, vitamin B_12_

## Abstract

The foodborne pathogen Listeria monocytogenes can form proteinaceous organelles called bacterial microcompartments (BMCs) that optimize the utilization of substrates, such as 1,2-propanediol, and confer an anaerobic growth advantage. Rhamnose is a deoxyhexose sugar abundant in a range of environments, including the human intestine, and can be degraded in anaerobic conditions into 1,2-propanediol, next to acetate and lactate. Rhamnose-derived 1,2-propanediol was found to link with BMCs in some human pathogens such as Salmonella enterica, but the involvement of BMCs in rhamnose metabolism and potential physiological effects on L. monocytogenes are still unknown. In this study, we first test the effect of rhamnose uptake and utilization on anaerobic growth of L. monocytogenes EGDe without or with added vitamin B_12_, followed by metabolic analysis. We show that vitamin B_12_-dependent activation of *pdu* stimulates metabolism and anaerobic growth of L. monocytogenes EGDe on rhamnose via 1,2-propanediol degradation into 1-propanol and propionate. Transmission electron microscopy of *pdu*-induced cells shows that BMCs are formed, and additional proteomics experiments confirm expression of *pdu* BMC shell proteins and enzymes. Finally, we discuss the physiological effects and energy efficiency of L. monocytogenes *pdu* BMC-driven anaerobic rhamnose metabolism and the impact on competitive fitness in environments such as the human intestine.

**IMPORTANCE**Listeria monocytogenes is a foodborne pathogen causing severe illness and, as such, it is crucial to understand the molecular mechanisms contributing to its survival strategy and pathogenicity. Rhamnose is a deoxyhexose sugar abundant in a range of environments, including the human intestine, and can be degraded in anaerobic conditions into 1,2-propanediol. In our previous study, the utilization of 1,2-propanediol (*pdu*) in L. monocytogenes was proved to be metabolized in bacterial microcompartments (BMCs), which are self-assembling subcellular proteinaceous structures and analogs of eukaryotic organelles. Here, we show that the vitamin B_12_-dependent activation of *pdu* stimulates metabolism and anaerobic growth of L. monocytogenes EGDe on rhamnose via BMC-dependent 1,2-propanediol utilization. Combined with metabolic and proteomics analysis, our discussion on the physiological effects and energy efficiency of BMC-driven rhamnose metabolism shed new light to understand the impact on L. monocytogenes competitive fitness in ecosystems such as the human intestine.

## INTRODUCTION

Listeria monocytogenes is a Gram-positive facultative anaerobe and a foodborne pathogen that causes a severe human infection called listeriosis (1, 2). The pathogen continues to cause foodborne illness outbreaks characterized by high mortality ranging from 20 to 30% (1, 3). L. monocytogenes is found ubiquitously in natural environments, and it can survive a variety of stress conditions leading to the colonization of different niches, including a range of food processing environments (1, 3, 4). To survive in such a variety of niches, L. monocytogenes should be able to adapt to environmental stresses and to use a range of nutrients for growth in aerobic and anaerobic conditions (1, 5, 6).

Recent studies on anaerobic growth of L. monocytogenes have provided evidence that it has the capacity to form proteinaceous organelles so-called bacterial microcompartments (BMCs) that enable extension of its metabolic repertoire by supporting the utilization of 1,2-propanediol and ethanolamine (7–9). BMCs are self-assembling organelles that consist of an enzymatic core that is encapsulated by a semipermeable protein shell (7, 10, 11). The separation of the encapsulated enzymes from the cytosol is thought to protect the cell from toxic metabolic intermediates such as aldehydes, and prevent unwanted side reactions (7, 10, 11). In our previous studies, we showed that the L. monocytogenes 1,2-propanediol utilization gene cluster (*pdu*) is activated in the presence of 1,2-propanediol and vitamin B_12_, resulting in stimulation of growth in anaerobic conditions (8). Vitamin B_12_ is required for activation of the *pdu* cluster in L. monocytogenes (8, 12) and to act as a cofactor of 1,2-propoanediol reductase (13). Activation of BMC-dependent *pdu* supports degradation of 1,2-propanediol via the toxic intermediate propionaldehyde into 1-propanol and propionate via respective reductive and oxidative branches, with the latter resulting in extra ATP generation leading to enhanced anaerobic growth of L. monocytogenes (8). Notably, 1,2-propanediol is a major end product from the anaerobic degradation of mucus-derived rhamnose by human intestinal microbiota, and it is thought to be an important energy source supporting the intestinal growth of selected pathogens such as Salmonella spp. and L. monocytogenes (7, 14–16).

Rhamnose is a naturally occurring deoxyhexose sugar abundant in glycans on surfaces of mammalian and bacterial cells and in the cell walls of many plant and insect species (14, 17). Anaerobic metabolism of rhamnose has been studied previously in a range of bacteria, including Escherichia coli, and rhamnose is parallelly metabolized into lactaldehyde and dihydroxyacetone phosphate (DHAP) (18, 19). DHAP is converted in the glycolytic pathway, leading to a variety of fermentation products, while lactaldehyde is converted to 1,2-propanediol that is subsequently secreted (18, 19). Notably, for example in Salmonella spp. and Clostridium phytofermentans, rhamnose-derived 1,2-propanediol can be converted to 1-propanol and propionate via BMC-dependent *pdu* (14, 16). Although rhamnose-derived 1,2-propanediol was found to be metabolized via a *pduD*-dependent pathway in Listeria innocua (20), the possible activation and contribution of BMC-dependent *pdu* to anaerobic metabolism and growth of L. monocytogenes on rhamnose remains to be investigated.

In this study, we first quantified the effect of rhamnose as sole carbon source on anaerobic growth and metabolism of L. monocytogenes in absence or presence of vitamin B_12_ (cobalamin), an essential cofactor of 1,2-propanediol reductase, the signature enzyme of BMC-dependent *pdu* (13). Next, we analyzed rhamnose utilization and end product formation and, combined with transmission electron microscopy (TEM) and proteomics, we provide evidence for a B_12_-dependent *pdu*-induced metabolic shift. We summarize our findings in a model integrating BMC-dependent *pdu* with rhamnose metabolism and discuss impact on growth and survival of L. monocytogenes in anaerobic environments such as the human intestine.

## RESULTS

### Activation of *pdu* stimulates anaerobic growth of *L. monocytogenes* EGDe on rhamnose.

**FIG 1:**
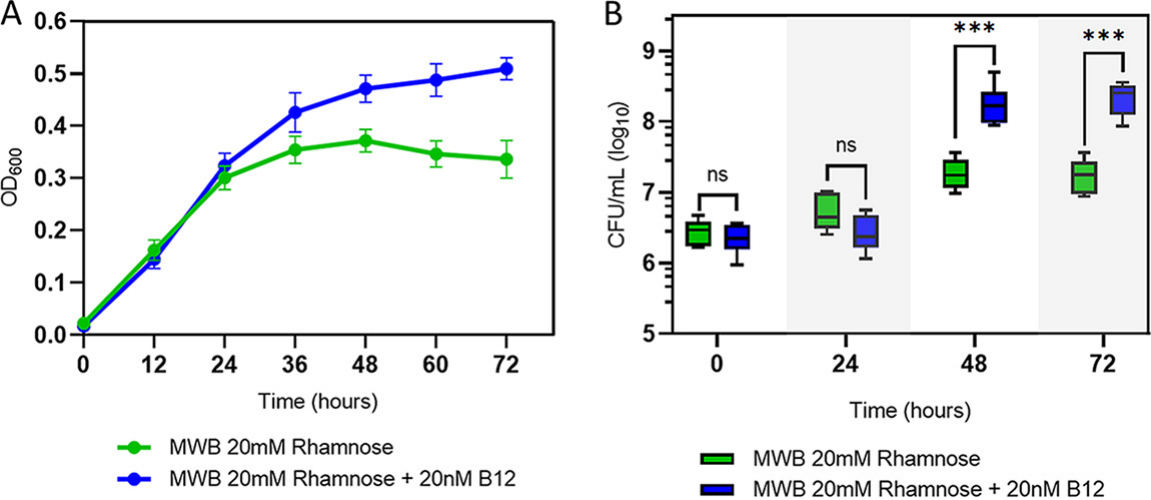
Impact of l-rhamnose and vitamin B_12_ on anaerobic growth of L. monocytogenes EGDe. (A) OD_600_ growth curves in MWB defined medium with 20 mM l-rhamnose as the sole carbon source (green symbols) and MWB with 20 mM rhamnose and 20n M B_12_ (blue symbols). (B) CFU during growth on MWB 20 mM rhamnose (green symbols) and on MWB 20 mM rhamnose plus 20 nM B_12_ (blue symbols). Results from three independent experiments with three technical repeats are expressed as mean and standard errors. Statistical significance is indicated (***, *P* < 0.001; ns, *P* > 0.05 [Holm-Sidak *t* test]).

We first examined whether rhamnose can function as a sole carbon source to support anaerobic growth of L. monocytogenes EGDe in MWB defined medium without or with added vitamin B_12_ (cobalamin) (Fig. 1). In MWB (modified Welshimer’s broth) defined medium supplied with 20 mM rhamnose, the optical density at 600 nm (OD_600_) reaches a maximum of about 0.37 after 48 h, while in MWB supplied with 20 mM rhamnose and 20 nM B_12_ OD_600_ continues to increase after 48 h, reaching a significantly higher OD_600_ of 0.51 at 72 h. Enhanced growth on MWB supplied with rhamnose and B_12_ compared to MWB plus rhamnose is also evident from plate counts, which increase from 6.5 to 8.2 log_10_ CFU/ml and from 6.5 to 7.2 log_10_ CFU/ml, respectively (Fig. 1B). There is no significant difference in growth performance of L. monocytogenes EGDe on MWB supplied with 20 mM glucose and MWB supplied with 20 mM glucose and 20 nM B_12_, and at 48 h final levels of 8.8 log_10_ CFU/ml were reached (see Fig. S1 in the supplemental material). These results suggest that B_12_-stimulated anaerobic growth of L. monocytogenes EGDe on MWB medium with rhamnose as the sole carbon source is linked to the activation of *pdu*.

10.1128/mSphere.00434-21.2 FIG S1 Anaerobic growth of L. monocytogenes EGDe on MWB plus glucose (green symbols) and MWB plus 20 mM glucose and 20 nM B_12_ (blue symbols). (A) OD_600_ growth curves; (B) CFU determined at indicated time points during growth on MWB plus 20 mM glucose (green bars) and MWB plus 20 mM glucose and 20 nM B_12_ (blue bars). Results from three independent experiments with three technical repeats are expressed as means and standard errors. Statistical significance is indicated (ns, *P* > 0.05 [Holm-Sidak *t* test]). Download FIG S1, TIF file, 1.1 MB. Copyright © 2021 Zeng et al. 2021 Zeng et al. https://creativecommons.org/licenses/by/4.0/ This content is distributed under the terms of the Creative Commons Attribution 4.0 International license.

### Activation of *pdu* supports 1,2-propanediol degradation and stimulates rhamnose metabolism.

**FIG 2:**
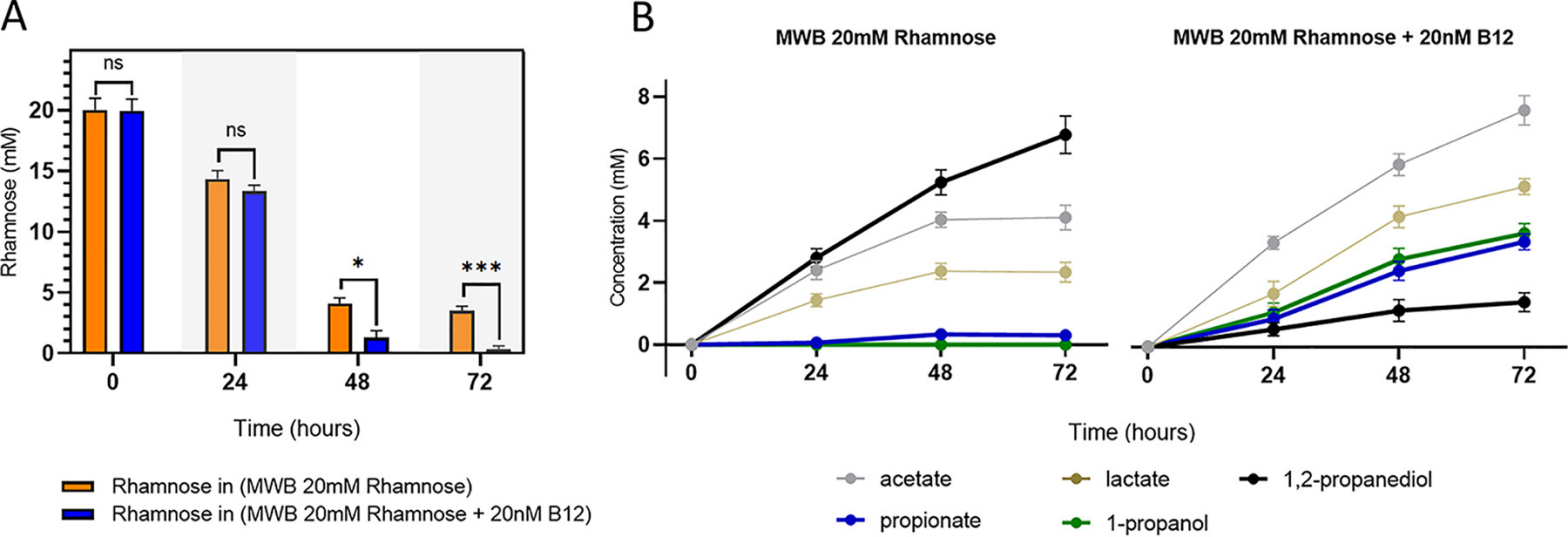
Impact of vitamin B_12_ on rhamnose metabolism of anaerobically grown L. monocytogenes EGDe. (A) Utilization of rhamnose by L. monocytogenes EGDe anaerobically grown in MWB plus 20 mM rhamnose (orange bars) and MWB plus 20 mM rhamnose and 20 nM B_12_ (blue bars). (B) Metabolites from rhamnose metabolism of L. monocytogenes EGDe anaerobically grown in MWB plus 20 mM rhamnose (left) and MWB plus 20 mM rhamnose and 20 nM B_12_ (right). Results from three independent experiments are expressed as means and standard errors. Statistical significance is indicated (***, *P* < 0.001; *, *P* < 0.05; ns, *P* > 0.05 [Holm-Sidak *t* test]).

To confirm possible activation of *pdu*, metabolic analysis via high-pressure liquid chromatography (HPLC) was conducted to quantify substrate consumption and product formation after anaerobic growth of L. monocytogenes EGDe on MWB plus 20 mM rhamnose and on MWB plus 20 mM rhamnose and 20 nM B_12_. As shown in Fig. 2A, at 72 h, the initial 20 mM rhamnose is completely consumed under a *pdu-*induced condition, whereas 3.5 mM rhamnose is retained under a *pdu*-noninduced condition. Additional end product analysis at 72 h shows the accumulation of ∼6.7 mM 1,2-propanediol under a *pdu*-noninduced condition and nearly zero production of propionate and 1-propanol. Under *pdu-*induced conditions, a significantly smaller amount of 1,2-propanediol is found, ∼1.4 mM, and higher levels of ∼3.4 mM propionate and 3.6 mM 1-propanol are produced at 72 h, in line with the expected 1:1 molar stoichiometry of L. monocytogenes BMC-dependent *pdu* (9). Enhanced rhamnose metabolism in *pdu*-induced cells is also evident from production of acetate and lactate. At 72 h, 4.1 mM acetate and 2.3 lactate are produced under *pdu-*noninduced conditions, while 7.6 mM acetate and 5.1 mM lactate are produced under *pdu-*induced conditions.

### Visualization of BMCs and expression analysis of BMC shell proteins.

**FIG 3:**
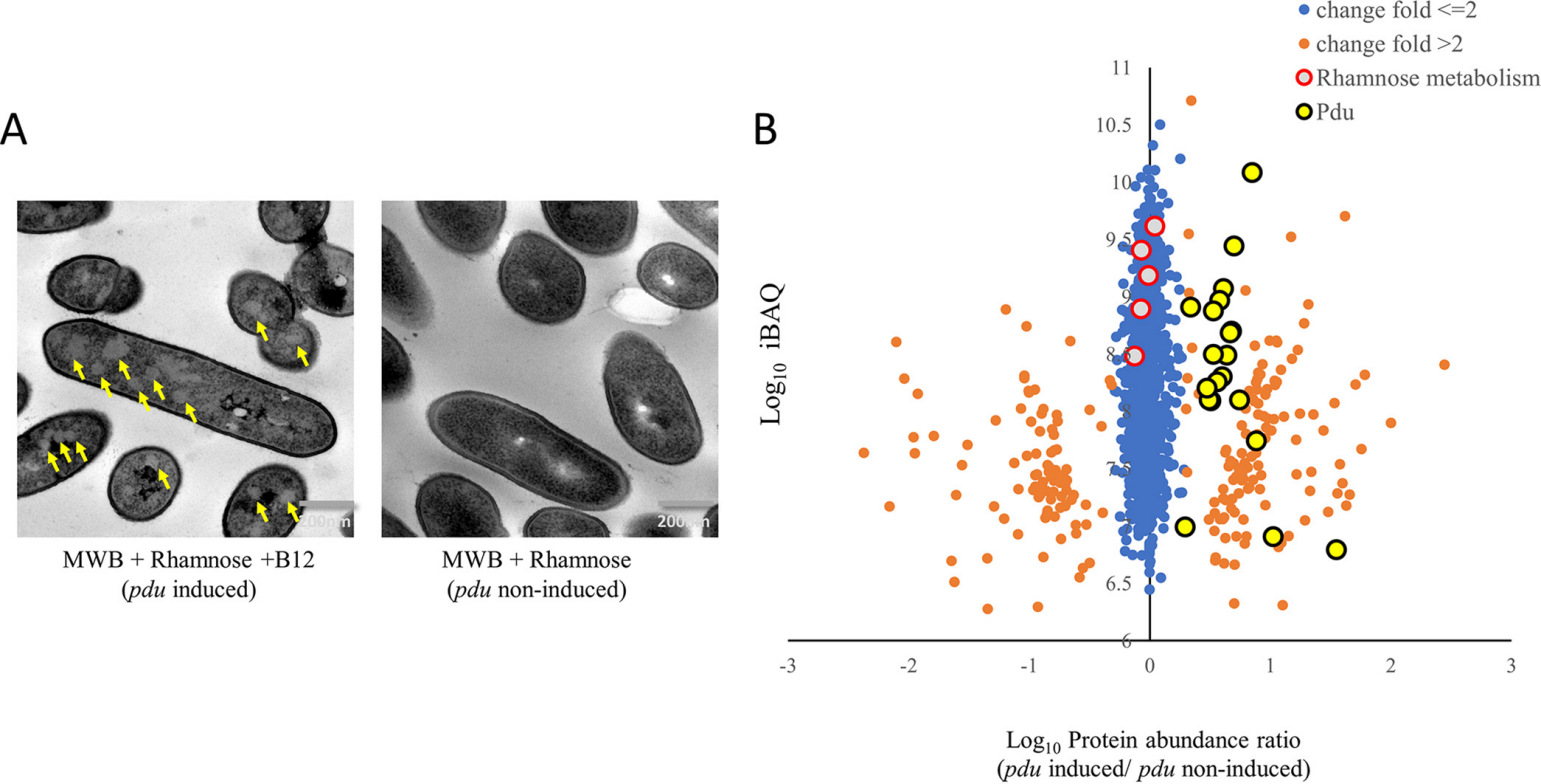
TEM visualization of BMCs and proteomics analysis of *pdu*-induced cells (MWB plus 20 mM rhamnose and B_12_) compared to *pdu*-noninduced cells (MWB plus 20 mM rhamnose). (A) TEM visualization of BMCs in cells grown on MWB plus 20 mM rhamnose and B_12_ (left; yellow arrows point to BMCs) and cells grown on MWB with 20 mM rhamnose (right). (B) Proteomic ratio plot of MWB plus 20 mM rhamnose and B_12_ compared to MWB plus 20 mM rhamnose-grown cells. Fold change ≤ 2 in blue, fold change > 2 in orange, proteins in the Pdu cluster are black encircled in yellow, and proteins in the rhamnose cluster are red-encircled in gray. More details are provided in the text and in Table S1.

To determine whether BMCs are formed to support the utilization of rhamnose-derived 1,2-propanediol, TEM was performed to observe BMCs structures, and proteomics was applied to measure the expression of BMC shell proteins (Fig. 3A). The *pdu*-induced cells clearly contain BMC-like structures (60 to 70% of 300 BMC-positive cells) with an approximate diameter of 50 to 80 nm, while similar structures were not observed in *pdu*-noninduced cells. Notably, the identified structures strongly resemble TEM pictures of previously reported *pdu* BMCs in L. monocytogenes (8, 9) and in S. enterica and E. coli (13, 21). Compared to *pdu*-noninduced cells, *pdu*-induced cells show significant upregulation of 21 measurable Pdu proteins (Fig. 3B), including seven proteins annotated as BMCs shell proteins, PduTUABKJN. Notably, *pdu*-induced and *pdu-*noninduced rhamnose-grown cells show similar expression of proteins in the rhamnose metabolism cluster (*lmo2850*, *rhaA*, *rhaB*, and *rhaM*) (Fig. 3B), which indicates that the activation of *pdu* BMC does not affect the expression of these enzymes.

10.1128/mSphere.00434-21.3 TABLE S1 Protein profiling of *pdu*-induced compared to *pdu*-noninduced L. monocytogenes EGDe in MWB medium with rhamnose. Download Table S1, XLSX file, 0.1 MB. Copyright © 2021 Zeng et al. 2021 Zeng et al. https://creativecommons.org/licenses/by/4.0/ This content is distributed under the terms of the Creative Commons Attribution 4.0 International license.

### Proteomics-based pathway visualization of propanoate metabolism and vitamin B_12_ metabolism.

**FIG 4:**
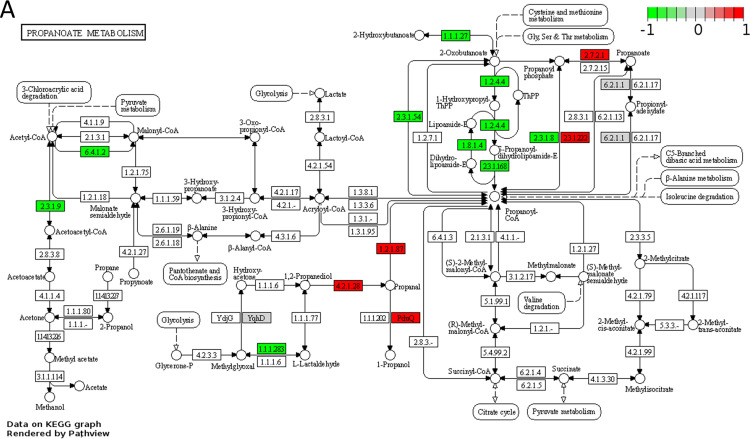

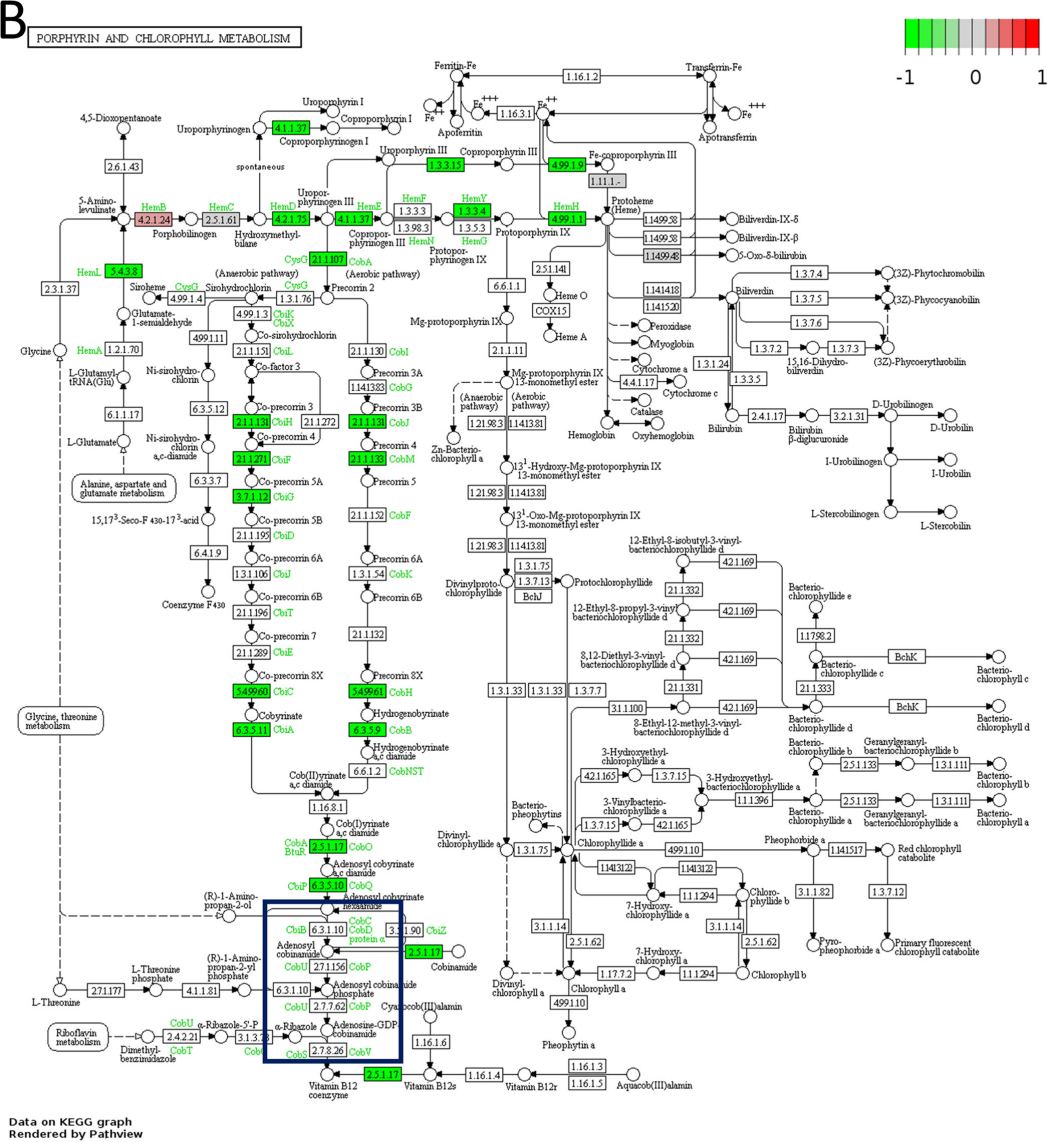
(A and B) Proteomics-based pathway visualization of propanoate metabolism (A) and porphyrin and chlorophyll metabolism (B) in *pdu*-induced compared to *pdu*-noninduced L. monocytogenes EGDe via Pathview. Rectangle boxes represent enzymes with the relative expression indicated based on proteomics data. Key metabolites are named, and the positions in the pathways are indicated by circles. In panel B, the blue box highlights B_12_ reactions that are encoded by the *pdu* cluster. More details are provided in the text and in Table S4.

To visualize the metabolism from 1,2-propanediol to propanoate (propionate) and 1-propanol, the identified proteins and expression levels presented in Table S1, are mapped to propanoate metabolic pathways of L. monocytogenes EGDe. As shown in Fig. 4A, the enzymes involved in degradation of rhamnose-derived 1,2-propanediol into propanoate (propionate) and 1-propanol are all significantly upregulated under *pdu*-induced conditions compared to *pdu*-noninduced conditions. The propanediol dehydratase (EC 4.2.1.28) is an enzyme with three subunits encoded by *pduC*, *pduD*, and *pduE*, which converts 1,2-propanediol into propanal (propionaldehyde). Propionaldehyde is metabolized to 1-propanol by propanol dehydrogenase PduQ and propanol coenzyme A (propanol-CoA) by propionaldehyde dehydrogenase PduP (EC 1.2.1.87). Propanol-CoA is converted to propanoyl-phosphate by phosphate propanoyltransferase PduL (EC 2.3.1.222), with propanoyl-phosphate subsequently converted to propanoate by propionate kinase PduW (EC 2.7.2.1). We found that the vitamin B_12_ biosynthesis pathway that is grouped in porphyrin and chlorophyll metabolism is significantly downregulated under *pdu*-induced conditions compared to *pdu*-noninduced conditions (proteomics-based pathway visualization of porphyrin and chlorophyll metabolism; Fig. 4B), which suggests that the supplementation of 20 nM B_12_ represses the expression of proteins required for B_12_ biosynthesis. This also includes the three enzymes mediating the final steps in B_12_ biosynthesis, CobU, CobS and CobC, encoded by the respective genes located in the *pdu* cluster (Fig. 4B) (8, 22–24). Apparently, B_12_ accumulation from the medium supports activation of *pdu* BMCs, whereas despite the expression of B_12_ biosynthesis enzymes, the production and levels of B_12_ reached are not sufficient to induce *pdu* in L. monocytogenes EGDe grown in MWB without added B_12_.

10.1128/mSphere.00434-21.6 TABLE S4 Annotation and proteins IDs of *rha* and *pdu* cluster. Download Table S4, XLSX file, 0.01 MB. Copyright © 2021 Zeng et al. 2021 Zeng et al. https://creativecommons.org/licenses/by/4.0/ This content is distributed under the terms of the Creative Commons Attribution 4.0 International license.

## DISCUSSION

**FIG 5:**
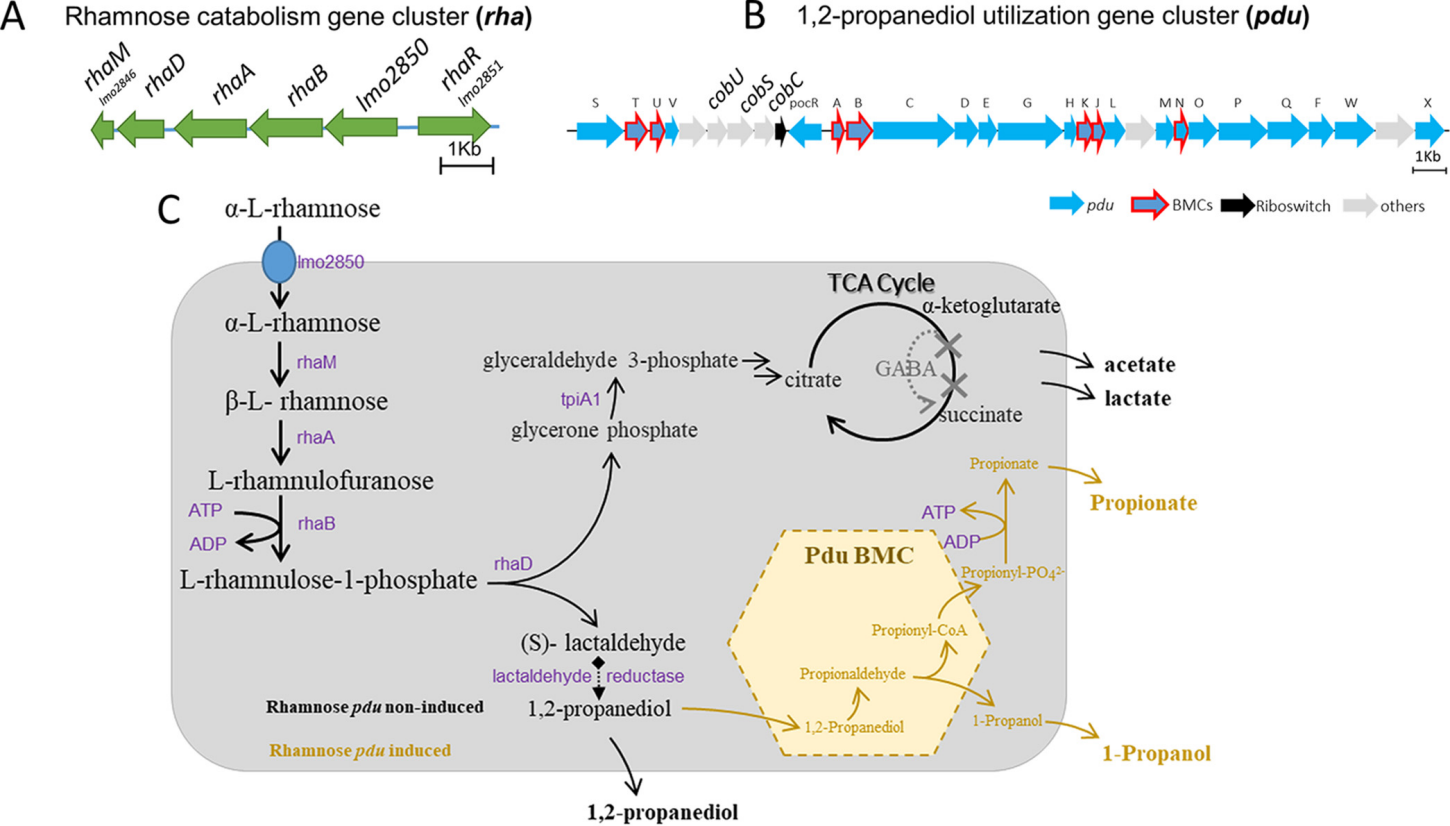
Overview of rhamnose metabolism with or without 1,2-propanediol BMCs in L. monocytogenes. (A) Rhamnose catabolism gene cluster, *rha*. (B) 1,2-Propanediol utilization gene cluster, *pdu*. Details for panels A and B are in Table S4. (C) Proposed rhamnose metabolism model based on this study. Arrows represent reactions and enzymes and compounds indicated in black represent rhamnose metabolism without BMCs, and 1,2-propanediol BMC reactions activated by B_12_ and compounds involved are shown in yellow. For details, see Results and Discussion.

The presented model of 1,2-propanediol BMCs in rhamnose metabolism is based on growth phenotypes, metabolic analysis, proteomics, TEM visualization, and our understanding of 1,2-propanediol BMCs in anaerobic growth of L. monocytogenes EGDe. As illustrated in Fig. 5, the rhamnose catabolism gene cluster (*rha*) in L. monocytogenes EGDe is composed of *lmo2846*-*lmo2851* (25). *lmo2850* encodes a secondary transporter which has high similarity with l-rhamnose permease RhaT in E. coli (26–28) and is conceivably acting as the transporter of α-l-rhamnose. l-Rhamnose mutarotase RhaM mediates the conversion of α-l-rhamnose into β-l-rhamnose (also called l-rhamnopyranose) (25, 29). β-l-Rhamnose is converted to l-rhamnulose by l-rhamnose isomerase RhaA (25, 30). l-Rhamnose is then phosphorylated to l-rhamnulose 1-phosphate by rhamnulokinase RhaB with one ATP consumption (25, 30). l-Rhamnulose 1-phosphate is split into (*S*)-lactaldehyde and dihydroxyacetone phosphate (DHAP) by rhamnulose-1-phosphate aldolase RhaD (25, 30). DHAP can be metabolized to glyceraldehyde 3-phosphate via triosephosphate isomerase 1 TpiA1 and, via the glycolytic pathway (14, 31) and the GABA (γ-aminobutyric acid) shunt in the incomplete tricarboxylic acid cycle in L. monocytogenes (32), to the end products acetate and lactate, as confirmed in our metabolic analysis. The observed production of 1,2-propanediol in *pdu*-noninduced conditions confirms the predicted anaerobic conversion of lactaldehyde to 1,2-propanediol in L. monocytogenes EGDe. The activity of lactaldehyde reductase has not been described in L. monocytogenes (33), but protein similarity alignment with lactaldehyde reductase FucO of Escherichia coli (33) suggests four putative candidates annotated as alcohol dehydrogenase in L. monocytogenes EGDe, including lmo1166, lmo1171, lmo1634, and lmo1737, detected in the proteomes of both *pdu*-noninduced and *pdu*-induced cells (for details see Text S1 in the supplemental material). Since the discovery of the role of *pdu* BMCs dehydratase in rhamnose (and fucose) utilization, two pathway scenarios have been proposed, one with and one without lactaldehyde reductase encapsulated inside BMCs (25, 29). In line with previously reported comparative genomic analysis (25, 29), our data now provide evidence for the latter model to be active in L. monocytogenes since rhamnose is converted via lactaldehyde to 1,2-propanediol in the absence of BMCs under the *pdu*-noninduced condition, while with added B_12_ the metabolism of 1,2-propanediol proceeds via *pdu* BMCs.

10.1128/mSphere.00434-21.1 TEXT S1 Protein similarity alignment with lactaldehyde reductase FucO of E. coli against L. monocytogenes EGDe. Download Text S1, TXT file, 0.002 MB. Copyright © 2021 Zeng et al. 2021 Zeng et al. https://creativecommons.org/licenses/by/4.0/ This content is distributed under the terms of the Creative Commons Attribution 4.0 International license.

The activation of *pdu* BMCs enhances anaerobic rhamnose metabolism in L. monocytogenes and conceivably generates additional energy via the ATP-producing propionate branch in *pdu* and via enhanced flux into the glycolytic pathway resulting in a significant stimulation of growth. At 72 h, 20 mM rhamnose is metabolized into 7.6 mM acetate, 5.1 mM lactate, 1.4 mM 1,2-propanediol, 3.4 mM propionate, and 3.6 mM 1-propanol under the *pdu*-induced condition, whereas 16.5 mM rhamnose is metabolized into 4.1 mM acetate, 2.3 mM lactate, and 6.7 mM 1,2-propanediol under the *pdu*-noninduced condition. The theoretical ATP yield from rhamnose conversion to lactate, acetate, and propionate includes the production of 1.5 ATP per 1 lactate, 2.5 ATP per 1 acetate, and 0.5 ATP per 1 propionate produced (for details of the reactions, see Table S3). Based on the concentrations of end products at 72 h, *pdu-*induced cells theoretically generate 1.425 ATP per 1 rhamnose, while *pdu-*noninduced cells generate 0.830 ATP per 1 rhamnose (for details on the calculations, see Table S3). The theoretical energy gain of L. monocytogenes EGDe from anaerobic rhamnose metabolism with the activation of 1,2-propanediol BMCs could offer an explanation for the 10-fold-higher number of CFU reached (8.2 log_10_ CFU/ml) compared to *pdu*-noninduced conditions (7.2 log_10_/ml).

10.1128/mSphere.00434-21.5 TABLE S3 Reaction list of rhamnose metabolism and theoretical ATP yield from rhamnose conversion to lactate, acetate, and propionate. Download Table S3, XLSX file, 0.01 MB. Copyright © 2021 Zeng et al. 2021 Zeng et al. https://creativecommons.org/licenses/by/4.0/ This content is distributed under the terms of the Creative Commons Attribution 4.0 International license.

Our data provide evidence for another extension of the BMC-dependent metabolic repertoire of L. monocytogenes under anaerobic conditions that now includes BMC-dependent ethanolamine utilization (*eut*) (9), BMC *pdu* (8), and BMC *pdu*-stimulated rhamnose metabolism. The indicated substrates can be found in a wide range of environments, including foods and the human gastrointestinal tract. Substrates for microcompartment metabolism such as ethanolamine and 1,2-propanediol are constantly produced in the human intestine by bacterial metabolism of food or host cell components. Enteric pathogens such as Salmonella spp. gain a competitive advantage in the intestine by utilizing these substrates, an advantage enhanced by the host inflammatory response (15, 34–36). It is conceivable that the competitive fitness of L. monocytogenes can be enhanced by activation of BMC-dependent *eut* and *pdu*, with corresponding substrates provided by enzymatic activities of gut microbiota, such as the release of ethanolamine following membrane phospholipid degradation and the release of rhamnose following mucus glycan hydrolysis activity, and propanediol as a fermentation product (15). Notably, despite the presence of a complete vitamin B_12_ synthesis cluster, we found that *eut* (9), *pdu* (8), and *pdu*-stimulated rhamnose utilization in L. monocytogenes in the present study requires supplementation of B_12_ to the medium. This points to an important role of B_12_ in activation of L. monocytogenes BMC-mediated metabolic pathways containing B_12_-dependent signature aldehyde reductases. Vitamin B_12_ can be found in foods, including meat and dairy products (23, 37), and is also found in human intestine, where part of the B_12_ is derived from gut microbiota that have the capacity to produce B_12_ (12, 23). The fact that in the present study we observed induction of the B_12_ synthesis pathway in cells grown in MWB plus rhamnose but no activation of B_12_-dependent *pdu*, whereas activation was found with B_12_ added to the medium, points to an intricate regulation of the B_12_ synthesis pathway and its connection to BMCs activation. In addition to earlier studies on transcriptional and translational control of BMC *eut* and *pdu* in L. monocytogenes (1, 12, 15, 23, 38), studies are required to assess for example impact of extracellular and intracellular B_12_ concentrations on activation of BMC pathways and their role in L. monocytogenes ecophysiology and virulence.

## MATERIALS AND METHODS

### Strains, culture conditions, and growth measurements.

All experiments in this study were carried out with L. monocytogenes EGDe anaerobically grown at 30°C in defined medium MWB (Modified Welshimer’s broth) (39). Overnight-grown cells in Luria broth (LB) were washed three times in phosphate-buffered saline before inoculation into MWB. MWB was supplemented with 20 mM l-rhamnose as the sole carbon source with or without the addition of 20 nM vitamin B_12_. Anaerobic conditions were achieved by using an Anoxomat anaerobic culture system with a gas mixture composed of 10% CO_2_, 5% H_2_, and 85% N_2_. MWB with 20 mM rhamnose and 20 nM vitamin B_12_ was defined as a rhamnose *pdu*-induced condition, while MWB with 20 mM rhamnose was defined as a rhamnose *pdu*-noninduced condition. OD_600_ measurements in MWB were performed every 12 h for 3 days. Plate counting in MWB to quantity the CFU was performed every 24 h for 3 days. All growth measurements were performed with three independent experiments with three technical repeats.

### Analysis of metabolites for rhamnose metabolism using HPLC.

Samples were taken from the cultures at 0, 24, 48, and 72 h. After centrifugation, the supernatant was collected for the HPLC measurements of rhamnose, acetate, lactate, 1,2-propanediol, 1-propanol, and propionate. The experiment was performed with three biological replicates. In addition, the standard curves of all the metabolites were measured in the concentrations 0.1, 1, 5, 10, and 50 mM. HPLC was performed using an Ultimate 3000 HPLC (Dionex) equipped with an RI-101 refractive index detector (Shodex, Kawasaki, Japan), an autosampler, and an ion-exclusion Aminex HPX-87H column (7.8 mm by 300 mm) with a guard column (Bio-Rad, Hercules, CA). As the mobile phase, 5 mM H_2_SO_4_ was used at a flow rate of 0.6 ml/min, and the column was kept at 40°C. The total run time was 30 min, and the injection volume was 10 μl. All HPLC measurements were performed with three independent experiments with three technical repeats.

### TEM.

L. monocytogenes EGDe cultures were grown anaerobically at 30°C under rhamnose *pdu*-induced or rhamnose *pdu*-noninduced conditions. Samples were collected at 48 h of incubation. About 10 μg of dry cells was fixed for 2 h in 2.5% (vol/vol) glutaraldehyde in 0.1 M sodium cacodylate buffer (pH 7.2). After a rinse in the same buffer, postfixation was done in 1% (wt/vol) OsO_4_ for 1 h at room temperature. The samples were dehydrated by ethanol and were then embedded in resin (Spurr HM20) for 8 h at 70°C. Thin sections (<100 nm) of polymerized resin samples were obtained with microtomes. After being stained with 2% (wt/vol) aqueous uranyl acetate, the samples were analyzed with a JEOL 1400 plus TEM at a 120-kV setting (8, 9). The observation of BMCs structures was performed within three biological replicates, and determination of the fraction of BMC-positive cells was based on the analysis of 300 cells in respective TEM pictures for both *pdu*-induced and *pdu*-noninduced conditions, as previously described (8).

### Proteomics.

L. monocytogenes cultures were anaerobically grown at 30°C under rhamnose *pdu*-induced and rhamnose *pdu*-noninduced conditions. Samples were collected at 48 h of incubation and then washed twice with 100 mM Tris (pH 8). About 10 mg (wet weight) of cells in 100 μl of 100 mM Tris was sonicated for 30 s twice to lyse the cells. Samples were prepared according to the filter-assisted sample preparation protocol (FASP) with the following steps: reduction with 15 mM dithiothreitol, alkylation with 20 mM acrylamide, and digestion with sequencing-grade trypsin overnight (40). Each prepared peptide sample was analyzed by injecting (18 μl) into a nano-LC-MS/MS (Thermo nLC1000 connected to a LTQ-Orbitrap XL) as described previously (8, 9). Liquid chromatography-mass spectrometry (LC-MS) data with all MS/MS spectra were analyzed with the MaxQuant quantitative proteomics software package as described before (8, 9, 41). A protein database with the protein sequences of L. monocytogenes EGDe (ID UP000000817) was downloaded from UniProt. Filtering and further bioinformatics and statistical analysis of the MaxQuant ProteinGroups file were performed with Perseus (42). Reverse hits and contaminants were filtered out. Protein groups were filtered to contain minimally two peptides for protein identification, of which at least one is unique and at least one is unmodified. A volcano plot was prepared based on the Student *t* test difference of a Pdu-induced versus a Pdu-noninduced control. The mass spectrometry proteomics data have been deposited to the ProteomeXchange Consortium via the PRIDE (43) partner repository with the data set identifier PXD025734 (https://www.ebi.ac.uk/pride/archive/projects/PXD025734).

### Bioinformatics and statistical analysis.

Pathview R package (44) to visualize the proteomics data: the UniProt protein IDs from Table S1 in the supplemental material were collected and retrieved to Entre IDs. A list of Entrez IDs, protein expression indicated by LFQ intensity (see Table S2), was mapped to the L. monocytogenes EGDe KEGG pathway database using the tool Pathview (R version 3.2.1). The box represents genes, and the different colors indicate the level of expression with default setting.

10.1128/mSphere.00434-21.4 TABLE S2 Input to Pathview with Entrez IDs and protein expression indicated by LFQ intensity. Download Table S2, XLSX file, 0.07 MB. Copyright © 2021 Zeng et al. 2021 Zeng et al. https://creativecommons.org/licenses/by/4.0/ This content is distributed under the terms of the Creative Commons Attribution 4.0 International license.

Statistical analyses were performed in Prism 8.0.1 for Windows (GraphPad Software). As indicated in the figure legends, statistical significances were determined using a Holm-Sidak *t* test and are indicated in the figures (***, *P* < 0.001; *, *P* < 0.05; ns, *P* > 0.05 ).

## References

[1] Radoshevich L, Cossart P. 2018. *Listeria monocytogenes*: towards a complete picture of its physiology and pathogenesis. Nat Rev Microbiol 16:32–46. doi:10.1038/nrmicro.2017.126. 29176582

[2] Jemmi T, Stephan R. 2006. *Listeria monocytogenes*: food-borne pathogen and hygiene indicator. Rev Sci Tech 25:571–580. doi:10.20506/rst.25.2.1681. 17094698

[3] Gahan CG, Hill C. 2014. *Listeria monocytogenes*: survival and adaptation in the gastrointestinal tract. Front Cell Infect Microbiol 4:9. doi:10.3389/fcimb.2014.00009. 24551601 PMC3913888

[4] NicAogáin K, O’Byrne CP. 2016. The role of stress and stress adaptations in determining the fate of the bacterial pathogen *Listeria monocytogenes* in the food chain. Front Microbiol 7:1865. doi:10.3389/fmicb.2016.01865. 27933042 PMC5120093

[5] Tompkin R. 2002. Control of *Listeria monocytogenes* in the food-processing environment. J Food Prot 65:709–725. doi:10.4315/0362-028x-65.4.709. 11952224

[6] Portman JL, Dubensky SB, Peterson BN, Whiteley AT, Portnoy DA. 2017. Activation of the *Listeria monocytogenes* virulence program by a reducing environment. mBio 8:e01595-17. doi:10.1128/mBio.01595-17. 29042499 PMC5646252

[7] Kerfeld CA, Aussignargues C, Zarzycki J, Cai F, Sutter M. 2018. Bacterial microcompartments. Nat Rev Microbiol 16:277–290. doi:10.1038/nrmicro.2018.10. 29503457 PMC6022854

[8] Zeng Z, Smid EJ, Boeren S, Notebaart RA, Abee T. 2019. Bacterial microcompartment-dependent 1, 2-propanediol utilization stimulates anaerobic growth of *Listeria monocytogenes* EGDe. Front Microbiol 10:2660. doi:10.3389/fmicb.2019.02660. 31803170 PMC6873790

[9] Zeng Z, Boeren S, Bhandula V, Light SH, Smid EJ, Notebaart RA, Abee T. 2021. Bacterial microcompartments coupled with extracellular electron transfer drive the anaerobic utilization of ethanolamine in *Listeria monocytogenes*. mSystems 6:e01349-20. doi:10.1128/mSystems.01349-20. 33850044 PMC8547011

[10] Yeates TO, Crowley CS, Tanaka S. 2010. Bacterial microcompartment organelles: protein shell structure and evolution. Annu Rev Biophys 39:185–205. doi:10.1146/annurev.biophys.093008.131418. 20192762 PMC3272493

[11] Liu LN. 2021. Bacterial metabolosomes: new insights into their structure and bioengineering. Microb Biotechnol 14:88–93. doi:10.1111/1751-7915.13740. 33404191 PMC7888463

[12] Mellin J, Tiensuu T, Bécavin C, Gouin E, Johansson J, Cossart P. 2013. A riboswitch-regulated antisense RNA in *Listeria monocytogenes*. Proc Natl Acad Sci U S A 110:13132–13137. doi:10.1073/pnas.1304795110. 23878253 PMC3740843

[13] Cheng S, Sinha S, Fan C, Liu Y, Bobik TA. 2011. Genetic analysis of the protein shell of the microcompartments involved in coenzyme B_12_-dependent 1, 2-propanediol degradation by Salmonella. J Bacteriol 193:1385–1392. doi:10.1128/JB.01473-10. 21239588 PMC3067621

[14] Petit E, LaTouf WG, Coppi MV, Warnick TA, Currie D, Romashko I, Deshpande S, Haas K, Alvelo-Maurosa JG, Wardman C, Schnell DJ, Leschine SB, Blanchard JL. 2013. Involvement of a bacterial microcompartment in the metabolism of fucose and rhamnose by *Clostridium phytofermentans*. PLoS One 8:e54337. doi:10.1371/journal.pone.0054337. 23382892 PMC3557285

[15] Jakobson CM, Tullman-Ercek D. 2016. Dumpster diving in the gut: bacterial microcompartments as part of a host-associated lifestyle. PLoS Pathog 12:e1005558. doi:10.1371/journal.ppat.1005558. 27171216 PMC4865037

[16] Obradors N, Badia J, Baldoma L, Aguilar J. 1988. Anaerobic metabolism of the l-rhamnose fermentation product 1, 2-propanediol in *Salmonella* Typhimurium. J Bacteriol 170:2159–2162. doi:10.1128/jb.170.5.2159-2162.1988. 3283105 PMC211101

[17] Tonetti M, Sturla L, Bisso A, Zanardi D, Benatti U, De Flora A. 1998. The metabolism of 6-deoxyhexoses in bacterial and animal cells. Biochimie 80:923–931. doi:10.1016/S0300-9084(00)88889-6. 9893952

[18] Chen Y, Tobin J, Zhu Y, Schleif R, Lin E. 1987. Cross-induction of the l-fucose system by l-rhamnose in *Escherichia coli*. J Bacteriol 169:3712–3719. doi:10.1128/jb.169.8.3712-3719.1987. 3301811 PMC212456

[19] Giraud M-F, Naismith JH. 2000. The rhamnose pathway. Curr Opin Struct Biol 10:687–696. doi:10.1016/s0959-440x(00)00145-7. 11114506

[20] Xue J, Murrieta CM, Rule DC, Miller KW. 2008. Exogenous or l-rhamnose-derived 1, 2-propanediol is metabolized via a *pduD*-dependent pathway in *Listeria innocua*. Appl Environ Microbiol 74:7073–7079. doi:10.1128/AEM.01074-08. 18805996 PMC2583486

[21] Dadswell K, Creagh S, McCullagh E, Liang M, Brown IR, Warren MJ, McNally A, MacSharry J, Prentice MB. 2019. Bacterial microcompartment-mediated ethanolamine metabolism in *E. coli* urinary tract infection. Infect Immun 87:e00211-19. doi:10.1128/IAI.00211-19. 31138611 PMC6652756

[22] Fang H, Kang J, Zhang D. 2017. Microbial production of vitamin B_12_: a review and future perspectives. Microb Cell Fact 16:1–14. doi:10.1186/s12934-017-0631-y. 28137297 PMC5282855

[23] Rowley CA, Kendall MM. 2019. To B_12_ or not to B_12_: five questions on the role of cobalamin in host-microbial interactions. PLoS Pathog 15:e1007479. doi:10.1371/journal.ppat.1007479. 30605490 PMC6317780

[24] Buchrieser C, Rusniok C, Consortium L, Kunst F, Cossart P, Glaser P, Listeria Consortium. 2003. Comparison of the genome sequences of *Listeria monocytogenes* and Listeria innocua: clues for evolution and pathogenicity. FEMS Immunol Med Microbiol 35:207–213. doi:10.1016/S0928-8244(02)00448-0. 12648839

[25] Fieseler L, Schmitter S, Teiserskas J, Loessner MJ. 2012. Rhamnose-inducible gene expression in *Listeria monocytogenes*. PLoS One 7:e43444. doi:10.1371/journal.pone.0043444. 22927968 PMC3425472

[26] Muiry J, Gunn T, McDonald T, Bradley S, Tate C, Henderson P. 1993. Proton-linked l-rhamnose transport, and its comparison with l-fucose transport in *Enterobacteriaceae*. Biochemical J 290:833–842. doi:10.1042/bj2900833. PMC1132357 8384447

[27] Glaser P, Frangeul L, Buchrieser C, Rusniok C, Amend A, Baquero F, Berche P, Bloecker H, Brandt P, Chakraborty T, Charbit A, Chetouani F, Couvé E, de Daruvar A, Dehoux P, Domann E, Domínguez-Bernal G, Duchaud E, Durant L, Dussurget O, Entian KD, Fsihi H, García-del Portillo F, Garrido P, Gautier L, Goebel W, Gómez-López N, Hain T, Hauf J, Jackson D, Jones LM, Kaerst U, Kreft J, Kuhn M, Kunst F, Kurapkat G, Madueno E, Maitournam A, Vicente JM, Ng E, Nedjari H, Nordsiek G, Novella S, de Pablos B, Pérez-Diaz JC, Purcell R, Remmel B, Rose M, Schlueter T, Simoes N, et al. 2001. Comparative genomics of *Listeria* species. Science 294:849–852. doi:10.1126/science.1063447. 11679669

[28] Rodionova IA, Li X, Thiel V, Stolyar S, Stanton K, Fredrickson JK, Bryant DA, Osterman AL, Best AA, Rodionov DA. 2013. Comparative genomics and functional analysis of rhamnose catabolic pathways and regulons in bacteria. Front Microbiol 4:407. doi:10.3389/fmicb.2013.00407. 24391637 PMC3870299

[29] Ryu K-S, Kim J-I, Cho S-J, Park D, Park C, Cheong H-K, Lee J-O, Choi B-S. 2005. Structural insights into the monosaccharide specificity of *Escherichia coli* rhamnose mutarotase. J Mol Biol 349:153–162. doi:10.1016/j.jmb.2005.03.047. 15876375

[30] Badía J, Baldomà L, Aguilar J, Boronat A. 1989. Identification of the *rhaA*, *rhaB*, and *rhaD* gene products from *Escherichia coli* K-12. FEMS Microbiol Lett 65:253–257. doi:10.1016/0378-1097(89)90226-7. 2558952

[31] Misra SK, Milohanic E, Aké F, Mijakovic I, Deutscher J, Monnet V, Henry C. 2011. Analysis of the serine/threonine/tyrosine phosphoproteome of the pathogenic bacterium *Listeria monocytogenes* reveals phosphorylated proteins related to virulence. Proteomics 11:4155–4165. doi:10.1002/pmic.201100259. 21956863

[32] Feehily C, O’Byrne CP, Karatzas KAG. 2013. Functional γ-aminobutyrate shunt in *Listeria monocytogenes*: role in acid tolerance and succinate biosynthesis. Appl Environ Microbiol 79:74–80. doi:10.1128/AEM.02184-12. 23064337 PMC3536111

[33] Cocks G, Aguilar J, Lin E. 1974. Evolution of l-1, 2-propanediol catabolism in *Escherichia coli* by recruitment of enzymes for l-fucose and l-lactate metabolism. J Bacteriol 118:83–88. doi:10.1128/jb.118.1.83-88.1974. 4595205 PMC246642

[34] Thiennimitr P, Winter SE, Winter MG, Xavier MN, Tolstikov V, Huseby DL, Sterzenbach T, Tsolis RM, Roth JR, Bäumler AJ. 2011. Intestinal inflammation allows *Salmonella* to use ethanolamine to compete with the microbiota. Proc Natl Acad Sci U S A 108:17480–17485. doi:10.1073/pnas.1107857108. 21969563 PMC3198331

[35] Sperandio V. 2018. Pathogens’ adaptation to the human host. Proc Natl Acad Sci U S A 115:9342–9343. doi:10.1073/pnas.1813379115. 30190426 PMC6156631

[36] Prentice MB. 2021. Bacterial microcompartments and their role in pathogenicity. Curr Opin Microbiol 63:19–28. doi:10.1016/j.mib.2021.05.009. 34107380

[37] Watanabe F, Yabuta Y, Tanioka Y, Bito T. 2013. Biologically active vitamin B_12_ compounds in foods for preventing deficiency among vegetarians and elderly subjects. J Agric Food Chem 61:6769–6775. doi:10.1021/jf401545z. 23782218

[38] Mellin J, Koutero M, Dar D, Nahori M-A, Sorek R, Cossart P. 2014. Sequestration of a two-component response regulator by a riboswitch-regulated noncoding RNA. Science 345:940–943. doi:10.1126/science.1255083. 25146292

[39] Schneebeli R, Egli T. 2013. A defined, glucose-limited mineral medium for the cultivation of *Listeria* spp. Appl Environ Microbiol 79:2503–2511. doi:10.1128/AEM.03538-12. 23377938 PMC3623203

[40] Wiśniewski JR, Zougman A, Nagaraj N, Mann M. 2009. Universal sample preparation method for proteome analysis. Nat Methods 6:359–362. doi:10.1038/nmeth.1322. 19377485

[41] Cox J, Hein MY, Luber CA, Paron I, Nagaraj N, Mann M. 2014. Accurate proteome-wide label-free quantification by delayed normalization and maximal peptide ratio extraction, termed MaxLFQ. Mol Cell Proteomics 13:2513–2526. doi:10.1074/mcp.M113.031591. 24942700 PMC4159666

[42] Bielow C, Mastrobuoni G, Kempa S. 2016. Proteomics quality control: quality control software for MaxQuant results. J Proteome Res 15:777–787. doi:10.1021/acs.jproteome.5b00780. 26653327

[43] Vizcaino JA, Csordas A, del-Toro N, Dianes JA, Griss J, Lavidas I, Mayer G, Perez-Riverol Y, Reisinger F, Ternent T, Xu QW, Wang R, Hermjakob H. 2016. 2016 update of the PRIDE database and its related tools. Nucleic Acids Res 44:11033–11033. doi:10.1093/nar/gkw880. 27683222 PMC5159556

[44] Luo W, Brouwer C. 2013. Pathview: an R/Bioconductor package for pathway-based data integration and visualization. Bioinformatics 29:1830–1831. doi:10.1093/bioinformatics/btt285. 23740750 PMC3702256

