# Supplementary figures and images for: Anaerobic Growth of Listeria monocytogenes on Rhamnose Is Stimulated by Vitamin B12 and Bacterial Microcompartment-Dependent 1,2-Propanediol Utilization
Source: mSphere. 2021 Jul 21;6(4):e00434-21. doi: 10.1128/mSphere.00434-21 (PMC8386454; doi:10.1128/mSphere.00434-21)

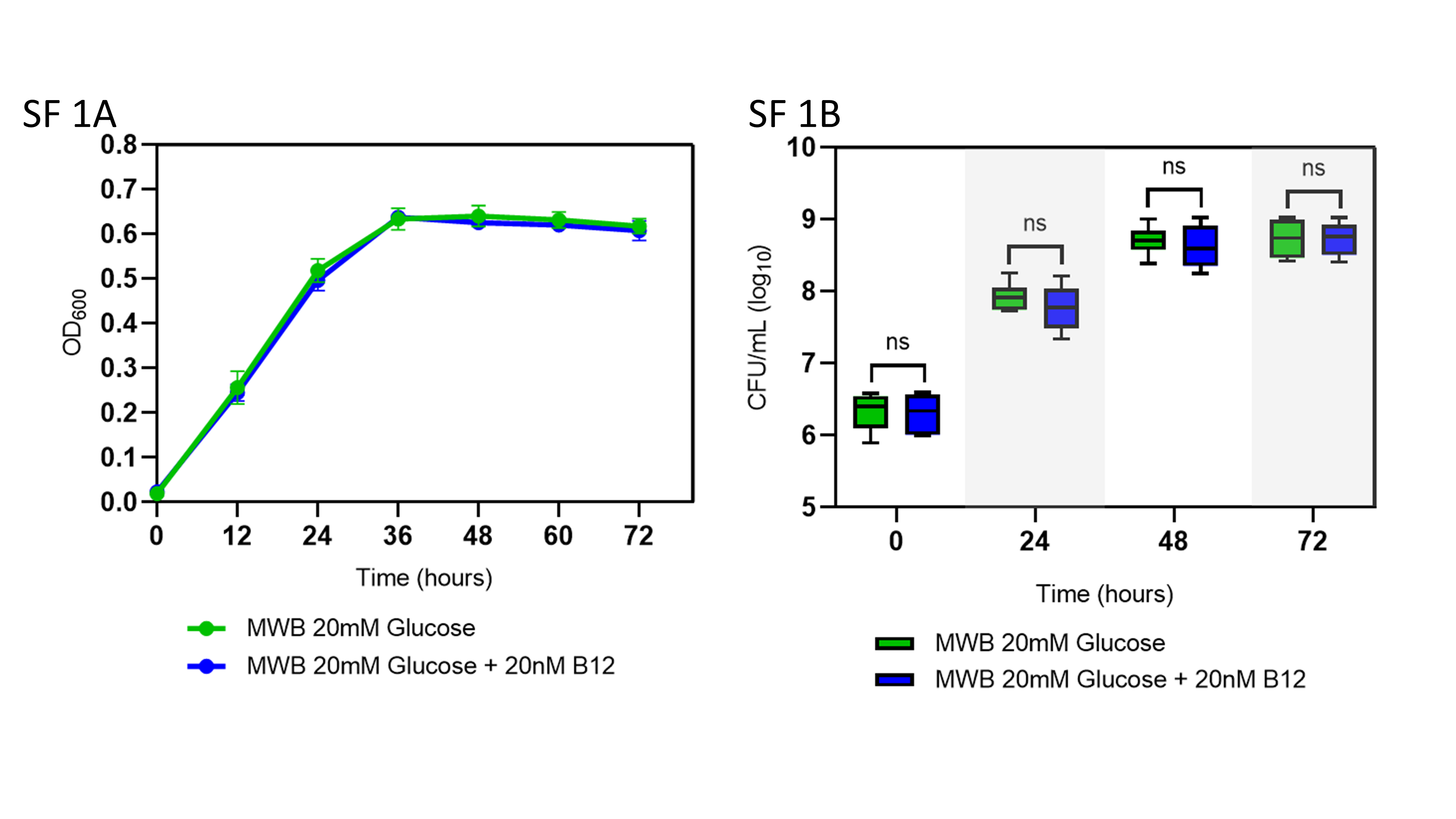

Supplement: FIG S1 [file msphere.00434-21-sf001.tif]
